# TIGIT-Fc as a Potential Therapeutic Agent for Fetomaternal Tolerance

**DOI:** 10.3389/fimmu.2021.649135

**Published:** 2021-03-25

**Authors:** Wenyan Fu, Renfei Cai, Zetong Ma, Tian Li, Changhai Lei, Jian Zhao, Shi Hu

**Affiliations:** ^1^ Department of Assisted Reproduction, Shanghai Ninth People’s Hospital, Shanghai Jiao Tong University School of Medicine, Shanghai, China; ^2^ Department of Biophysics, College of Basic Medical Sciences, Second Military Medical University, Shanghai, China; ^3^ Team SMMU-China of International Genetically Engineered Machine (iGEM) Competitions, Department of Biophysics, Second Military Medical University, Shanghai, China; ^4^ KOCHKOR Biotech, Inc., Shanghai, China

**Keywords:** TIGIT, targeted therapy, fetomaternal tolerance, RAS, IgG based therapy

## Abstract

The perfect synchronization of maternal immune-endocrine mechanisms and those of the fetus is necessary for a successful pregnancy. In this report, decidual immune cells at the maternal-fetal interface were detected that expressed TIGIT (T cell immunoreceptor with Ig and ITIM domains), which is a co-inhibitory receptor that triggers immunological tolerance. We generated recombinant TIGIT-Fc fusion proteins by linking the extracellular domain of TIGIT and silent Fc fragments. The treatment with TIGIT-Fc of human decidual antigen presenting cells (APCs), the decidual dendritic cells (dDCs), and decidual macrophages (dMϕs) increased the production of interleukin 10 and induced the decidua APCs to powerfully polarize the decidual CD4^+^ T cells toward a classic T_H_2 phenotype. We further proposed that Notch signaling shows a pivotal effect on the transcriptional regulation in decidual immune cell subsets. Moreover, the administration of TIGIT-Fc to CBA/J pregnant mice at preimplantation induced CD4^+^ forkhead box P3^+^ (Foxp3^+^) regulatory T cells and tolerogenic dendritic cells and increased pregnancy rates in an abortion-prone animal model stress. The results suggested the therapeutic potential of the TIGIT-Fc fusion protein in reinstating immune tolerance in failing pregnancies.

## Introduction

A successful pregnancy is a unique type of immunological process in which the semiallogeneic paternal antigens carried by the fetus are accepted by the maternal immune system, allowing trophoblasts to invade. Meanwhile, the defense mechanisms against pathogens in the maternal immune system are preserved. However, the mechanisms regulating these unique immunological behaviors and maintaining the harmonious coexistence of maternal- and fetal-derived cells remain poorly understood (1). Given that the dysregulation of maternal–fetal immunity and deficient placentation have a notable relationship with pregnancy loss and pregnancy complications, such as fetal growth restriction (FGR) (2), recurrent pregnancy loss (RPL) (3), and pre-eclampsia (PE) (4), further studies to advance the diagnosis and prevention of these conditions are urgently needed.

Up to 5% of all women attempting to conceive are affected by PRL, which is defined as two or more miscarriages (5). During the past few decades, growing evidence has proven the inevitable role of a misdirected maternal immune response in PRL. Because of the disturbance of hematological and immunological homeostasis, both autoimmune diseases and alloimmune disorders can create a uterine microenvironment that is difficult for the embryo and invading conceptus-derived placental trophoblasts. Disappointingly, current immunotherapies for PRL, including the use of hormones, antithrombotic drugs, intralipids, intravenous immunoglobulin (IVIG), cytokine agonists or antagonists, and allogeneic lymphocytes, have not consistently yielded successful pregnancy outcomes (6).

T cell immunoreceptor with Ig and ITIM domains (TIGIT, also known as Vstm3, VSIG9, and WUCAM) is a member of the immunoglobulin superfamily and belongs to the poliovirus receptor (PVR)/nectin family. Structurally, the N terminus of TIGIT is an extracellular immunoglobulin variable-set (IgV) domain, which is followed by a transmembrane domain and an intracellular domain. The intracellular domain of TIGIT contains a canonical immunoreceptor tyrosine-based inhibitory motif (ITIM) and an immunoglobulin tyrosine tail (ITT) motif (7). TIGIT was first identified in a genomic search for genes that encoded potential inhibitory receptors, which were identified according to the presence of certain protein domain structures that were expressed specifically in T cells (7). TIGIT expression is strictly limited to lymphocytes and shows the highest expression in follicular helper CD4^+^ T cells, effector and regulatory CD4^+^ T cells, effector CD8^+^ T cells, and natural killer (NK) cells (7–12). PVR, also known as CD155, Necl5, and Tage4, was identified as a cognate receptor for TIGIT with high affinity. Despite their weaker affinities, PVRL3 and CD112 (also known as PVRL2/nectin 2) were also shown to bind to TIGIT (7). A ‘lock and key’ trans-interaction between the TIGIT IgV domain and cis-homodimers of PVR was mediated by the distinctive (V/I)(S/T)Q, AX6G, and T(F/Y)PX1G submotifs (7, 9, 13), which define the PVR/nectin family comprising TIGIT, CD226, CD96, CD112R, PVR, CD112, and CD113 (also known as PVRL3/nectin 3) (7, 13–16). Nectins and nectin-like proteins are a group of surface receptors that function through homophilic and heterophilic trans-interactions and consequently mediate cell–cell adhesion, cell polarization, tissue organization, and signal transduction (17, 18).

Recently, an increasing number of mechanisms underlying TIGIT immune suppression have been identified. TIGIT can not only inhibit natural killer (NK) cell effector function but also suppress their dendritic cell costimulatory ability. The former blocks initial target cell death and the release of cancer-related antigens, and the latter results in increases in anti-inflammatory cytokines such as IL-10 and reduced target cell antigen presentation.

TIGIT could also stimulate PVR signaling on other cells, such as tumor cells. Suppressed CD8^+^ T cell effector function or skewed CD4^+^ T cell polarization could be provoked by TIGIT, PVR-stimulated myeloid cells, and TIGIT^+^ regulatory T cells (Tregs), which can also inhibit CD8^+^ T cells and prevent the elimination of target cells (19).

Previously, we showed that the administration of the TIGIT-Fc fusion protein to NZB/W F1 mice decreased the production of anti-double-stranded DNA antibodies, alleviated proteinuria and prolonged survival compared with that in mice treated with control IgG. The TIGIT-Fc fusion protein showed an IgG-like stability that was similar to that of CTLA-4-Fc. Here, we found that TIGIT was also expressed by decidual immune cells at the maternal-fetal interface during early pregnancy. The TIGIT-Fc fusion protein with silent Fc fragments guided dDCs to strongly polarize decidual CD4^+^ T cells toward a classic T_H_2 phenotype. In a mouse model, we obtained new experimental evidence to support the administration of TIGIT-Fc to promote fetomaternal tolerance and demonstrated the therapeutic potential of TIGIT-Fc to restore immune tolerance in failing pregnancies.

## Material and Methods

### Primary Human Cells, Cell Lines and Reagents

PBMCs and decidual samples were isolated from the same patients of clinically normal pregnancies, which were terminated for nonmedical reasons (first-trimester, 7–12 wk gestation, n = 20) and 15 miscarriages (7–8 wks gestation, n = 15), which were classified as unexplained after the exclusion of maternal anatomic or hormonal abnormalities, or paternal and maternal chromosomal abnormalities. All specimens were collected by using a protocol approved by the Second Military Medical University Review Board, and written informed consent was obtained from each donor.

Pieces of decidual tissue were homogenized and digested with 0.5% collagenase type IV/20% heat-inactivated fetal calf serum overnight, or 2% collagenase type IV for 1 h at room temperature with gentle rotation. A single cell suspension was obtained by passing the supernatant through a series of cell separators to 40 mm and then layering the cells and performing density gradient separation with the standard Ficoll-Hypaque method, as reported previously (20, 21). T cells, NK cells and T cell subsets were further sorted by flow cytometry with different makers: CD3 (HIT3a), CD4 (SK3), CD45RA (L48), CD45RO (UCHL-1), and CD56 (B159). CD14^+^ macrophages were sorted by magnetic beads (Miltenyi Biotec). Decidual DC (dDC) were sorted use previous reported immunofluorescence label (22) (CD3^-^CD14^-^CD56^-^CD19^-^HLA-DR^+^) (CD19: SJ25C1, CD14: MφP9, HLD-DR: TU36). All antibodies are from BD Biosciences. Decidual NK cells (dNK) were further sorted as previous reported marks (CD45^+^ CD14^−^ CD3^−^CD56^bright^) (23) before analysis. For all subsets, at least 98% purity were confirmed based on reanalysis immediately after sorting. 0.5% BSA and 2% normal fetal bovine serum in PBS were used as blocking reagent. JEG-3 cells and HTR-8/SVneo were purchased from the American Type Culture Collection (ATCC, Manassas, VA). The identities of the cell lines were verified by STR analysis, and the cell lines were confirmed to be mycoplasma free. The cells were maintained in DMEM/1640 medium with 10% fetal bovine serum. Cell culture media and supplements were obtained from Life Technologies, Inc. LPS was purchased from Sigma. A FDA-approved Drug Library of 360 compounds was purchased from Selleck.

### Real-Time Polymerase Chain Reaction (PCR)

Total RNA was isolated with the RNeasy Mini Kit (Qiagen) according to the manufacturer’s instructions. Real-time quantitative PCR was performed with an ABI PRISM 7900HT instrument with the commercially available TaqMan probes Hs00545087 (*TIGIT*), Hs00197846 (*CD155*), Hs01071562 (*CD112*) and Mm03807522 (*Tigit*). Data were normalized to β-actin (24), which served as an endogenous control, and analyzed using SDS v2.3 (Applied Biosystems).

### Flow Cytometry

Cell surface staining was performed for 30 min at 4°C and was analyzed using a FACSCalibur flow cytometer (BD Biosciences) and CellQuest Software (BD Biosciences). Cellular staining was performed for 60 min on ice after using a fixation/permeabilization kit (eBioscience). A minimum of 1 × 10^4^ events were examined. The experiments were repeated independently three times with similar results.

### Fusion Proteins

As previously reported (7, 25), a recombinant plasmid was constructed by fusing the Fc segment of human IgG1 or murine IG2a, encoding the hinge-CH2-CH3 segment, to the C-termini of the extracellular domains (ECDs) of human and murine TIGIT, respectively. The LALA-PG Fc variant was constructed as previously described (26). All fusion proteins were obtained *via* the FreeStyle 293 expression system (Invitrogen) according to previously reported methods (27, 28) and subsequently purified using protein A-sepharose from the harvested cell culture supernatant. The purity of the fusion protein was determined by polyacrylamide gel electrophoresis. The protein concentration was measured according to the UV absorbance at a wavelength of 280 nm.

### Affinity Measurement

By using standard amine-coupling chemistry, we immobilized an anti-murine Fc polyclonal antibody (Jackson ImmunoResearch Europe Ltd.) on a CM5 chip (~150 RU) by using a previously reported method (25). The measurement of the monovalent binding affinity of the fusion protein was calculated by using surface plasmon resonance (SPR) (BIAcore-2000).

### IgG Biological Effect Assays

For the *in vitro* ADCC assay, SupT1 cells expressing murine PVR and A431 cells (high-expressing PVR cells) were labeled with 5 mM carboxyfluorescein succinimidyl ester (CellTrace CFSE Cell Proliferation Kit, Life Technologies) and co-cultured with murine or human macrophages overnight, respectively, at the indicated ratios in the presence of TIGIT-Fc fusion proteins.

For C1q ELISA, an ELISA sandwich-type immunoassay was used to analyze the binding of the different fusion proteins to C1q. Each fusion protein was coupled to a hydrophobic Maxisorp 96-well plate at eight different concentrations between 10 and 50 μg/ml. After washing, the C1q samples were incubated on the plate to allow C1q to bind to the fusion proteins. The bound C1q molecules were further washed and detected by anti-C1q antibodies followed by an HRP-labeled secondary antibody.

### Animal Studies

*In vivo* experiments were approved by the Institutional Animal Care and Use Committee (IACUC) of Second Military Medical University. C.B-17SCID; DBA/2J and CBA/J mice were provided by the Animal Center of the Second Military Medical University. All animals were treated in accordance with the guidelines of the Committee on Animals of the Second Military Medical University. The pharmacokinetic parameters (PK) of the fusion proteins were determined in female C.B-17 SCID mouse models. The fusion proteins were administered to eight-week-old mice at a dose of 1 mg/kg body weight by tail vein injection. Blood was collected in heparin-containing tubes and centrifuged to obtain the plasma samples. The serum concentration of the fusion proteins was determined by ELISA.

For the drug treatment studies, all mice were used at 10–12 weeks of age. To explore the protective role of TIGIT-Fc during pregnancy, an immunological model of abortion was used in which DBA/2J-mated CBA/J females were randomized and divided into different treatment groups. The day of vaginal plug formation was taken as day 0.5 of coitus. Selected mice were treated with 20 mg/kg fusion proteins or control IgG (i.v.) on 1.5 and 3.5 day postcoitum (dpc). Mated females were killed on 6.5 dpc and Paraaortic lymph nodes (PALN) and uterus cells were analyzed. Thereafter, 8 mice per group were killed at 12 dpc to assess the pregnancy and abortion rates.

For the single cell suspensions from PALN, the tissue was carefully squeezed through a 40-µm nylon cell strainer and washed with PBS. After washing, the cell suspension was filtered a second time with a 40-μm cell strainer. For the preparation of uterus cells, briefly, uteri were collected, cut into small pieces, and digested for 30 min at 37°C in HBSS buffer containing 1 mg/mL collagenase, 0.5 mg/mL hyaluronidase, 0.2 mg/mL DNase I, and 1 mg/mL BSA. HBSS buffer solution containing uterus cells was passed through a 100-μm strainer (SPL), and suspended cells were collected and washed. To analyze the Foxp3^+^ regulatory T-cell population, isolated uterine and PALN cells were first incubated with an anti-CD16/32 antibody (eBioscience) to block Fc receptors (FcRs), followed by incubation with anti-CD4 and anti-CD25 antibodies. Cells were then fixed, washed, permeabilized and incubated with anti-Foxp3 antibody. FcR-blocked uterus cells were incubated with antibodies against CD11c, MHC-II, and CD80 to characterize uterine DC phenotypes. All antibodies are from eBioscience.

### Cytokine Analysis and Multiplex Bead Array

Cytokine analysis was also performed on supernatants from indicated treatment using a human cytokine 10-plex panel (Thermo Scientific) per the manufacturer’s instructions, and read on a Luminex Analyzer. For the detection of IL-10, IL-12, IL-4, IL-5, TNF and IFN-γ, the supernatant of cells was harvested and measured according to the manufacturer’s instructions (Biolegend).

### Compound Library Screen

The established co-cultured cells were screened against 360 compounds from an FDA-approved drug library (SelleckChem, Houston, USA) to identify potent TIGIT enhancer. Co-cultured cells were plated in 96-well plates and treated with vehicle (0.01% DMSO) or the compound library (average compound concentration of the library in medium was 10 µM). After a one-day incubation with the drug library, TIGIT expression was detected by qPCR.

### Statistical Analysis

Unless otherwise specified, Student’s t test was used to evaluate the significance of the differences between two groups, and ANOVA was used to evaluate differences among three or more groups. Differences between samples were considered statistically significant when P < 0.05.

## Results

### TIGIT Is Expressed on Primary Human Decidual Lymphocyte Cells

**Figure 1:**
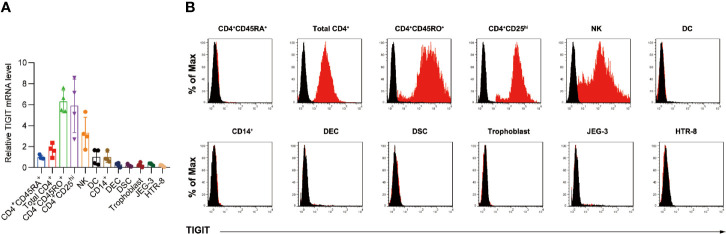
Expression of TIGIT protein in decidual immune cells. **(A)**, qPCR of the expression of TIGIT mRNA in total CD4^+^, CD4^+^CD45RO^+^, CD4^+^CD45RA^+^, CD4^+^CD25hi, NK, DC, CD14^+^ monocyte/macrophages, decidual epithelial cells (DECs), decidual stromal cells (DSCs), trophoblasts, JEG-3, and HTR-8/SVneo cells relative to TIGIT expression in naive CD4^+^CD45RA^+^ cells. Data are mean ± s.d. of four independent biological replicates. **(B)**, Membrane-bound TIGIT expression in different subsets of immune cells. The expression of TIGIT on different human immune cells was detected by staining with the indicated antibody, followed by flow cytometry analysis. The histograms shown in black correspond to the isotype controls, whereas the red histograms indicate the positive fluorescence (*n* = 4 independent biological experiments with similar results).

We first detected TIGIT expression by quantitative RT-PCR in decidual immune cell subsets from early pregnancy (**Figure 1a**). The high expression of TIGIT was detected in CD4^+^CD25^hi^ T cells, memory CD45RO^+^ cells and dNK cells, while naive CD45RA^+^ T cells, DC cells and decidual CD14^+^ monocytes/macrophages showed low levels of TIGIT mRNA expression (**Figure 1A**). Moreover, we detected very low TIGIT expression in decidual epithelial cells or decidual stromal cells, as well as the cell line JEG-3 and HTR-8/SVneo. Further flow cytometry analysis confirmed that TIGIT expression was absent in decidual CD45RA^+^ CD4^+^ T cells and CD11c^+^ DC cells and was highest in CD4^+^CD25^hi^ Treg cells and CD45RO^+^ T cells (**Figure 1B**).

The expression of CD155 and CD112, the TIGIT functional receptors, was also evaluated (**Figure S1**). Both CD155 and CD122 are extensively expressed in trophoblastic cells, decidual epithelial cells, or decidual stromal cells. In decidual immune cell subsets, both CD155 and CD122 are highly expressed in the decidual CD14^+^ monocytes/macrophages and DC cells, while very low expressed in T cells and NK cells.

### TIGIT-Fc Modifies Decidual APC Cytokine Production and Polarized Decidual CD4^+^ T Cells Toward a T_H_2 Phenotype

To investigate the therapeutic potential of TIGIT, we developed and generated a fusion protein by linking the extracellular domain of human TIGIT to the human IgG1 Fc region (hTIGIT-Fc_wt). For the possibility of investigation of such recombinant fusion protein in mouse models, a recombinant protein counterpart of murine TIGIT fused with murine IgG2a Fc chain (mTIGIT-Fc_wt) was also developed in our study. The antibody Fc region regulates the antibody serum half-life and cytotoxic activity. As the TIGIT functional receptor, PVR, is ubiquitously expressed in the human placenta as showed in our study and previously report (29). Within the relevant therapeutic context, the cytotoxicity of an antibody is not desirable and can lead to safety issues by initiating native host immune defenses against cells with receptor antigen expression. Therefore, we used LALA-PG Fc variants (hTIGIT-Fc_ LALA-PG; mTIGIT-Fc_ LALA-PG) that block complement binding and fixation as well as Fc-γ-dependent, antibody-dependent, and cell-mediated cytotoxity caused by both murine IgG2a and human IgG1. As previously reported, the fusion proteins showed high affinity for binding to CD155 (**Figure S2A**). We also found that hTIGIT-Fc bound to murine CD155, but such binding could not be detected between mTIGIT-Fc and human CD155 (**Figure S1A**). We next assessed the capacity of these fusion proteins to deplete PVR-expressing cells co-cultured with monocyte-derived macrophages *in vitro* at various effector to target (E: T) cell ratios (**Figures S2B** and **C**). As predicted, the hTIGIT-Fc_wt or mTIGIT-Fc_wt fusion proteins demonstrated strong ADCC activity, while the Fc-silent LALA-PG proteins showed negligible effects. In the C1q binding assays, only fusion proteins with the wild-type Fc showed remarkable binding to the C1q protein, which is part of the complement cascade (**Figures S2D** and **E**). All LALA-PG protein variants were devoid of any detectable binding at protein concentrations of up to 50 μg/ml. In comparison with the Fc fusion protein CTLA4-Fc, which has been well studied in previous studies, the TIGIT-Fc fusion proteins exhibited IgG-like stability and a similar denaturation temperature. The lowest concentrations (< 2%) of low molecular weight and high molecular weight products were observed after storage at 1 mg/mL at 40°C for 3 weeks (**Table S1**). A single intravenous dose of TIGIT-Fc proteins and CTLA4-Fc were separately administered to mice to measure the pharmacokinetic (PK) parameters. The main PK parameters of TIGIT-Fc proteins and CTLA4-Fc were very similar in mice and indicated the advantageously high stability of the TIGIT-Fc fusion proteins (**Table S1**). These data show that the hTIGIT-Fc_LALA-PG and mTIGIT-Fc_LALA-PG Fc variants do not induce any FcγR- or complement-mediated effector functions. Therefore, we used these proteins in the following experiments.

**Figure 2:**
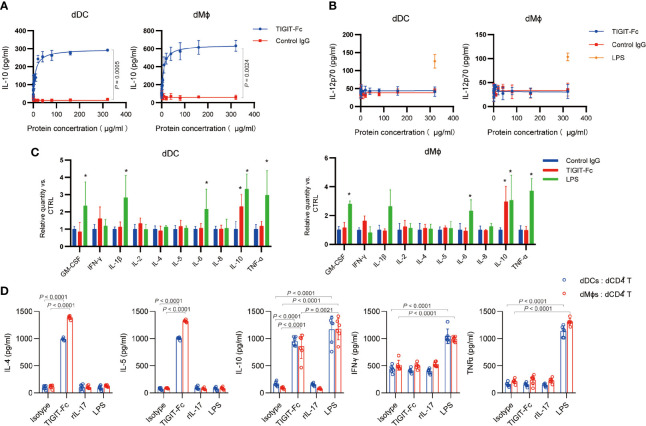
Immunomodulatory effect Biological effect of TIGIT fusion proteins *in vitro*. **(A)**, ELISA of IL-10 in human dDCs and dMϕs after the indicated treatment for 48 h with different concentration of TIGIT-Fc or control IgG, *n* = 4. **(B)**, ELISA of IL-2 in human dDCs and dMϕs after the indicated treatment for 48 h with different concentration of TIGIT-Fc or control IgG. LPS served as a positive control. *n* = 4. **(C)**, Cytokine levels in the supernatants of human dDCs and dMϕs were determined by multiplex bead array. The relative level was calculated as the ratio to the control IgG treatment, *n* = 4. **(D)**, The human dDCs and dMϕs were treated with TIGIT-Fc, IL-7, or LPS, respectively, for 24 h, washed and cocultured with decidual CD4^+^ T (dCD4^+^ T) cells. Thereafter, the T cells were transferred to a new 96-well round-bottom plate precoated with anti-CD3 and anti-CD28 (2 μg/ml each) and cultured for 24 h. The cytokine secretion of the CD4^+^ T cells was then determined by ELISA, *n* = 6. Data are the means ± s.d. **(A–D)**. *P* values were from a nonparametric t test (Mann-Whitney test) **(A, B)**, and two-way ANOVA followed by the Bonferroni post-test **(C, D)**. *: *P* < 0.05 **(C)**.

A previous report has shown that treatment with TIGIT-Fc during monocyte-derived DC maturation influenced DC cytokine production (7). In decidua, both dDCs and decidual macrophages are reported to be APCs and have tolerant effect; therefore, we tested whether TIGIT-Fc with a silent IgG has effect on the cytokine production of the sorted human decidual APCs form normal pregnancies. Interestingly, treatment of TIGIT-Fc significantly induced the production of IL-10 in a dose-dependent manner, with a half maximal effective concentration (EC50) of 8.336 μg/ml for dDCs and 7.085 μg/ml for dMϕs (**Figure 2A**). Conversely, treatment of TIGIT-Fc did not affect the secretion of IL-12p70, even at the highest dose used in our study, whereas 100 ng LPS treatment significant induce IL-12p70 protein production (**Figure 2B**). In order to further assess the immune-regulatory potential of TIGIT, we measured cytokines in supernatants from treated cells by multiplex bead array (**Figure 2C**). Following TIGIT-Fc treatment in dMϕ, we observed notable increased secretion of the cytokine IL-10. No discernable patterns could be confidently drawn with GM-CSF, IL-1β, IL-2, IL-4, IL-5, IL-6, IL-8, IFN-γ and TNF-α. In contrast to TIGIT-Fc treatment, we observed increased induction of GM-CSF, IL-1β, IL-6, IL-10, and TNF-α by LPS treatment. We also found increased secretion of the cytokine IL-10, but not IFN-γ in dDCs, further suggesting an immune- tolerance role of TIGIT.

Next, we investigated whether TIGIT-Fc-induced dMϕ and dDCs could polarize decidual CD4^+^ T cells toward a T_H_2 phenotype. Dϕs or dDCs pretreated with TIGIT (10 μg/mL), IL-7 (100 ng/mL), or LPS (100 ng/mL) for 48 h were cocultured with decidual CD4^+^ T cells for 3 days, respectively. IL-4, IL-5, IL-10, IFN-γ, and TNF-α in the supernatant were measured by ELISA (**Figure 2D**). Our data show that when Dϕs and dDCs were cocultured with decidual CD4^+^ T cells, IL-4 IL-5 and IL-10, but not IFN-γ and TNF-α, were markedly upregulated by TIGIT-Fc treatment, compared with that in decidual CD4^+^ T cells cocultured with dDCs but without TIGIT-Fc. However, we did not observe similar phenomena for IL-7- or LPS-activated dDCs or Dϕs, indicating that TIGIT-F-stimulated decidual APCs can polarize decidual CD4^+^ T cells toward a T_H_2-biased profile. These results indicated that treatment with TIGIT-Fc could influence the T_H_1/T_H_2 balance at the maternal-fetal interface.

The binding of TIGIT-Fc have the potential to block TIGIT- mediated signaling in T cells though its ITT and ITIM motifs. Interestingly, TIGIT-Fc or anti-TIGIT treatment (etigilimab) had no effect on T cell proliferation induced by a range of concentrations of plate-bound anti-CD3 & anti-CD28 of human decidual CD4^+^CD45RO^+^ T cells, which had high expression of TIGIT (**Figure S3A**). Moreover, TIGIT-Fc or anti-TIGIT treatment had no effect on cytokine production (**Figure S3B**). These data suggest that TIGIT-Fc had negligible effect on dCD4+ T cells alone and that TIGIT-Fc may regulate T cell activation by interacting with APCs

### Notch Signaling Is Critical for TIGIT Expression

**Figure 3:**
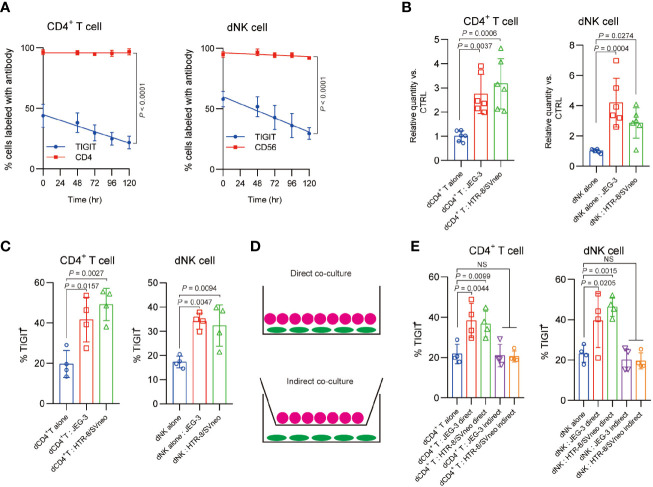
Direct TIGIT expression regulation of immune cells by trophoblast cells. **(A)**, The proportions of cells with positive TIGIT expression determined by flow cytometry analyses in different time are shown. CD4 and CD56 were served as a control marker for dCD4^+^ cells (left) and CD56 (right), *n* = 4. **(B)**, qPCR of the expression of human *TIGIT* mRNA in CD4^+^ T and NK cells, which were cultured alone or with different trophoblasts, *n* = 6. **(C)**, The proportions of cells with positive TIGIT expression determined by flow cytometry analyses in CD4^+^ T and NK cells, which were cultured alone or with different trophoblasts, *n* = 4. d, Schematic view of different co-culture models. **(E)**, The proportions of cells with positive TIGIT expression determined by flow cytometry analyses in dCD4+ T and dNK cells alone or cultured with different trophoblasts in different models, *n* = 4. Data are the means ± s.d and *P* values were from a one-way ANOVA followed by Tukey post-test **(A–D)**.

In our experiments, we found a notable time-dependent decrease of TIGIT expression in human dCD4^+^ T cells and dNK cells during *in vitro* culture alone (**Figure 3A**). Interestingly, co-culture CD4^+^ T cells and dNK cells with trophoblast cell line JEG-3 and HTR-8/SVneo significantly increased the expression level of TIGIT mRNA compared with mono-culture alone, with a 2.75 and 3.12-fold in dCD4^+^ T cells, respectively, and that in dNK cells by 4.2- and 2.8-fold, respectively (**Figure 3B**). Further flow cytometry analysis verified these results and suggested an enhanced effect of trophoblast cell on TIGIT expression (**Figure 3C**). As indirectly co-culture trophoblast cells with dCD4^+^ T cells and dNK cells (**Figures 3D, E**) did not have any effect on the TIGIT expression, we hypothesis a direct cell-to-cell interaction mechanism underlie the regulation of TIGIT expression.

**Figure 4:**
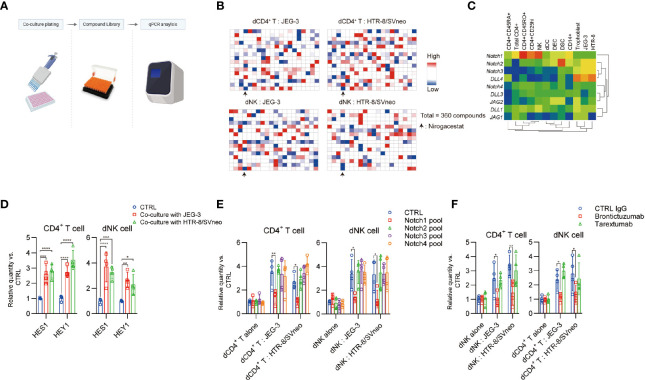
Notch signaling is involved for TIGIT expression in decidual cells. **(A)**, Schematic view of drug screen. Figure created with BioRender.com **(B)**, Heat map generated from the transcript expression TIGIT as determined by quantitative polymerase chain reaction (qPCR) analysis. In order for the data to usefully predict antagonism or sensitivity, a criterion that only dates in the upper and down quarters was employed for hit selection. **(C)**, Heat map representing transcript expression of Notch receptors and ligands, as determined by qPCR analysis. **(D)**, dCD4^+^ T and dNK cells were cultured alone or co-cultured with indicated cells and select gene expression was determined by qPCR analysis. Gene expression was normalized to the housekeeping gene and expressed as fold of control cells, *n* = 4. **(E)**, dCD4^+^ T and dNK cells were transfected with a CTRL pool (CTRL-siRNA) and a pool of Notch siRNAs. After 48 h, the cells were then cultured in different methods with indicated treatment for 24 h days, and TIGIT expression was determined by qPCR analysis. Gene expression was normalized to the housekeeping gene and expressed as fold of control cells cultured alone, *n* = 4. **(F)**, dCD4^+^ T and dNK cells were cultured alone or co-cultured with indicated cells and treated with different antibodies. TIGIT expression was determined by qPCR analysis. Gene expression was normalized to the housekeeping gene and expressed as fold of control cells cultured alone, *n* = 4. Data are the means ± s.d and *P* values were from a two-way ANOVA followed by the Bonferroni post-test **(D–F)**. *: *P* < 0.05; **: *P* < 0.01 **(E, F)**.

A functional screening method was used to identify potent TIGIT expression enhancer by treatment of dCD4^+^ T cells and dNK cells with a FDA-approved drug library (360 compounds), as described in **Table S1**. In the trophoblast cell line co-culture system, different compounds were administered and TIGIT expression was measured using qPCR assay (**Figure 4A**). Then, positive or negative impacts of different compounds on the TIGIT expression were calculated (**Figure 4B**). Nirogacestat was listed on all the inhibiting candidate lists of the dCD4^+^ T cells, dNK cells co-cultured with different trophoblast cell lines. This drug suggest the Notch signaling involved in the regulation of TIGIT expression induced by co-culture. Mammals have four Notch paralogues (Notch1–4) and various ligands in the Delta-like (DLL1, DLL3, and DLL4) and Jagged (JAG1 and JAG2) protein families. Interestingly, a high level expression of DLL4 was detected in both human primary trophoblast cells and trophoblast cell lines. Notch1 expression was notably higher than other notch receptors in dCD4^+^ T cells and dNK cells (**Figure 4C**). Real-time PCR analysis of the expression of Notch target genes *HES1* and *HEY1* showed a notable increase in dCD4^+^ T cells and dNK cells after co-culture with trophoblast cell lines (**Figure 4D**).

In addition, although the decidual immune cell expressed the Notch1 to Notch4 receptors, only treatment with Notch1 small interfering RNA (siRNA) pools, but not Notch2, 3 and 4 siRNA pools, effectively inhibited co-culture induced TIGIT expression (**Figure 4E**). Administration of Notch1 blocking antibody brontictuzumab, but not Notch2 & 3 targeting antibody tarextumab, significantly inhibited the co-culture induced TIGIT expression (**Figure 4F**). These results indicated that a curial role of the Notch1 receptors in the maternal-fetal interface could lead to TIGIT expression in the decidual immune cells.

### Low TIGIT Expression at the Maternal-Fetal Interface From Miscarriage

We next investigated TIGIT expression between the normal human pregnancy and miscarriage tissue. Notably, a qPCR analysis of the TIGIT mRNA in the placenta shows that early normal pregnancy presents significantly higher TIGIT than that of the unexplained miscarriage (**Figure S4A**). Moreover, the frequency of TIGIT positive dCD4 T cells and dNK cells from the normal early pregnancy is significantly higher than that of the miscarriage (**Figure S4B**).

### TIGIT Reduces Fetal Resorption in a Mouse Model

**Figure 5:**
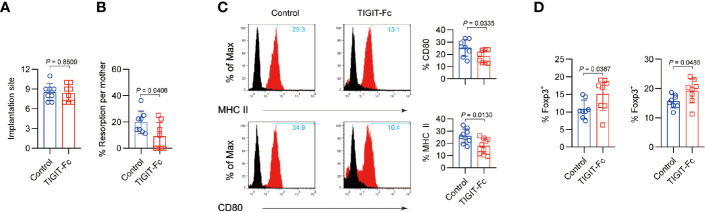
Administration of TIGIT-Fc protects fetuses from abortion. Treatment with mTIGIT-Fc of pregnant CBA/J females mated with DBA/2J males at 1.5 and 3.5 dpc. **(A)**, the total number of implantation sites and **(B)**, Resorption rates were measured at 12.5 dpc in the mated female mice, *n* = 8. **(C)** Phenotypic analysis of CD11c^+^ uterine DCs from mice treated with control IgG (Control) or TIGIT-Fc at 6.5 dpc. The percentages of MHC-II- and CD80-positive cells are plotted as bar graphs (right panels), *n* = 8. **(D)** Percentages of CD4^+^Foxp3^+^ T cells in the uterus (left) and PALNs (right) of different groups were calculated by flow cytometry analysis at 6.5 dpc. *n* = 8. Data are the means ± s.d **(A–D)** and all the *P* values were from a one-way ANOVA followed by Tukey post-test **(A–D)**.

We further used a well-established CBA/J×DBA/2J abortion-prone mouse model of pregnancy failure to explore the role of mTIGIT-Fc in fetomaternal tolerance. In the model, an unchanged the number of fetal implantations (**Figure 5A**) but a reduced incidence of fetal resorption (**Figure 5B**) was observed in the DBA/2J-mated CBA/J females administered mTIGIT-Fc. The phenotype of decidua CD11c^+^ cells was detected for further examination of the inhibitory effects of TIGIT-Fc on the maturation of CD11c^+^ cells. There was a notable decrease of maturation of uterine CD11c^+^ cells, and it showed suppressed levels of MHC-II and CD80 in abortion-prone animals treated with mTIGIT-Fc when compared to vehicle-treated animals. (**Figure 5C**). Additionally, both the uterus and PALNs of mTIGIT-Fc-treated female mice showed a higher proportion of Foxp3^+^ cells at 6.5 dpc than that of control females (**Figure 5D**). To further test whether TIGIT-Fc has a direct impact on the intrinsic function of the receptor, we assessed the cytokine production induced by anti-CD3 and anti-CD28 in sorted mice decidual CD4^+^ cells, but found no evidence of T cell inhibition (**Figure S5**). Overall, the results of our study indicate that TIGIT-Fc protects embryos from maternal immune rejection by inducing tolerant DCs (tDCs) and Foxp3^+^ cells.

## Discussion

Accumulating evidence has shown an immune modulating role of TIGIT in the context of autoimmunity and cancer. We previously evaluated the therapeutic role of TIGIT-Fc in a model of murine lupus. However, in that study, the TIGIT-Fc protein we used was a fusion protein containing the murine TIGIT-ECD linked to the murine IgG2a chain without any modification of the Fc domain. In this study, we used a LALA-PG Fc variant to eliminate potential cytotoxicity. This is important because decidual APC, dDCs, and dMϕs, which are key players in maternal immune tolerance, express high levels of the TIGIT functional receptor CD155. Moreover, a TIGIT-Fc with a wild-type Fc domain shows a strong effect on ADCC and C1q binding activity *in vitro*, further verifying the need to re-engineer the molecular structure of the TIGIT therapeutic protein.

We found that TIGIT is expression by decidual lymphocyte cell subsets, consistent with previous reports (30). Since CD155 is a receptor extensively expressed on human trophoblasts and decidual cells, it is not surprising that TIGIT participates in the development of normal human early pregnancy. The extravillous trophoblasts are in close contact with resident APCs in the decidua, usually in the decidua basalis (31). Moreover, our data show that high levels of secreted IL-10 were observed when decidual APCs were treated with TIGIT-Fc, endowing decidual APCs with the ability to induce the total decidual CD4^+^ T cells to produce increased levels of IL-5, IL-4, and IL-10 and minimal inflammatory cytokines, such as TNF-α and IFN-γ. A previous report showed that TIGIT inhibited the killing of PVR-expressing target cells by primary NK cells (32) and by immortalized YTS NK cells (33), we also determined whether TIGIT-Fc could block the intrinsic receptor signaling of TIGIT in T cells, operating by signaling downstream of its ITT and ITIM motifs. However, we were unable to demonstrate a direct effect of TIGIT-Fc in dCD4+ T cells, suggesting that the immune tolerant effect of TIGIT-Fc is dependent on APCs. Moreover, our data also showed that TIGIT was decreased in the dCD4^+^ or dNK cells from miscarriage patients, indicating that TIGIT down-regulation induced dysfunction of the APCs may contribute to the disease. Interestingly, we observed that TIGIT expression is down-regulated during the *in vitro* culture of decidual immune cells, indicating a conditionally expression pattern of TIGIT. Direct, but not indirect, co-culture of trophoblast with decidual immune cells significantly enhances TIGIT expression, suggesting a cell-to-cell contact based signaling regulation of TIGIT. Using a compound library, we identified that Notch signaling is involved in the transcriptional regulation of TIGIT in dCD4^+^ T cells and dNK cells. This is of interest because Notch signaling itself is depended on a cell-to-cell pattern: engagement of the Notch receptor with its ligand—delta family proteins that are presented on the surface of partner cells—leads to intramembrane proteolysis [sequential proteolysis by adisintegrin and metalloproteinase (ADAM) metalloprotease and the gamma-secretase complex (34)]. The induced cleavage of the receptor releases the intracellular fragment of Notch. Moreover, the TIGIT-CD155 signaling is also based on the cell-to-cell contact, suggesting a potent crosstalk of those two pathways between trophoblast and decidual immune cells. TIGIT-Fc may be an effective treatment for recurrent miscarriage, especially for those with TIGIT dysregulation or low expression. Moreover, the pharmacokinetics of TIGIT has been investigated in mice. Thus, preclinical study and clinical study are expected to examine the efficacy of TIGIT-Fc in recurrent miscarriage.

Here, recurrent miscarriage case showed a low expression of TGIT; however, heterogeneity has also been observed. Novel biomarkers associated with treatment outcome are therefore needed to be identified. Notably, we provide evidence that intervening the process of immune-related abortion with TIGIT-Fc can be achieved, but our *in vivo* efficacy models may not fully recapitulate human RAS, and the data are from a small number of animals. Moreover, the mechanisms responsible for these therapeutic effects of TIGIT-Fc are currently not well characterized.

## Conclusions

Overall, the immunoregulatory role of TIGIT-Fc may provide insights into the regulation mechanisms of maternal immunity that allow successful pregnancies. Moreover, our data on the immunoregulatory therapeutic efficiency of TIGIT provided new data and information for understanding the function of fusion protein treatment under pathogenic conditions. TIGIT-Fc-based bio-therapy is expected to be a potent approach for the treatment of recurrent miscarriage with an immune etiology.

## Data Availability Statement

The raw data supporting the conclusions of this article will be made available by the authors, without undue reservation.

## Ethics Statement

The studies involving human participants were reviewed and approved by Second Military Medical University Review Board. The patients/participants provided their written informed consent to participate in this study. The animal study was reviewed and approved by Institutional Animal Care and Use Committee (IACUC) of Second Military Medical University.

## Author Contributions

All authors contributed to the research design. WF, ZM, TL, CL, and SH performed research. WF, RC, CL, JZ and SH analyzed the data. WF, CL and SH wrote the paper. All authors contributed to the article and approved the submitted version

## Funding

This study was financially supported by the National Natural Science Foundation of China (grant no. 81773261, 31970882, 81903140 and 81602690); Shanghai Rising-Star Program (19QA1411400); Shanghai Sailing Program (19YF1438600); Shanghai Chenguang Program (17CG35); Shanghai Biomedical Technology Support Project (20S11906600) and the Open Project Grant from Engineering Research Center of Cell & Therapeutic Antibody, Ministry of Education, of Shanghai JiaoTong University.

## Conflict of Interest

The authors declare the following competing interests: JZ is a shareholder at KOCHKOR Biotech, Inc., Shanghai. WF and SH are inventors on intellectual property related to this work.

The remaining authors declare that the research was conducted in the absence of any commercial or financial relationships that could be construed as a potential conflict of interest.
