# Supplementary material for: TIGIT-Fc as a Potential Therapeutic Agent for Fetomaternal Tolerance
Source: Front Immunol. 2021 Mar 25;12:649135. doi: 10.3389/fimmu.2021.649135 (PMC8027249; doi:10.3389/fimmu.2021.649135)
Supplement: Supplementary file 1 [file DataSheet_1.pdf]

Supplementary Information for:
TIGIT-Fc as a Potential Therapeutic Agent for Fetomaternal Tolerance

Contents **Page**

Supplementary Figures..2

Figure S1. Expression of TIGIT function receptors in decidual cells. 2

Figure S2. Characterization of TIGIT fusion proteins...3

Figure S3. Direct Effect of TIGIT-Fc on human decidua T cells..4

Figure S4. Higher TIGIT expression at maternal-fetal interface in the early pregnancy than miscarriage. 5

Figure S5. Direct Effect of TIGIT-Fc on mice decidua T cells...6

Supplementary Tables 7

Table S1. Selected analytical data and pharmacokinetic parameters of recombinant fusion proteins in mice...7

Table S2. Details of compound library in each plate 8

Supplementary Figures

Figure S1

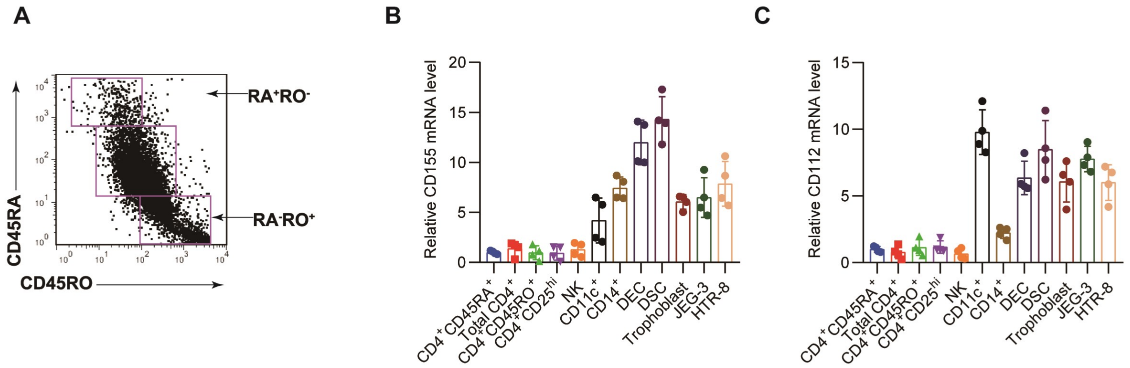

Figure S1. Expression of TIGIT function receptors in decidual cells. (A) Gating strategies used for flow cytometry analysis of CD4$^+$CD45RO$^+$, CD4$^+$CD45RA$^+$ cells. Real-time PCR of the expression of CD155**(B)** and CD112 **(C)** mRNA in total CD4$^+$, CD4$^+$CD45RO$^+$, CD4$^+$CD45RA$^{+,}$ CD4$^+$CD25^{hi}, NK, DC, CD14$^+$ monocyte/macrophages, decidual epithelial cells (DECs), decidual stromal cells (DSCs), trophoblasts, JEG-3 and HTR-8/SVneo cells relative to the expression of that in naive CD4$^+$CD45RA$^+$ cells, Data are mean ± s.d. of four independent biological replicates.

Figure S2

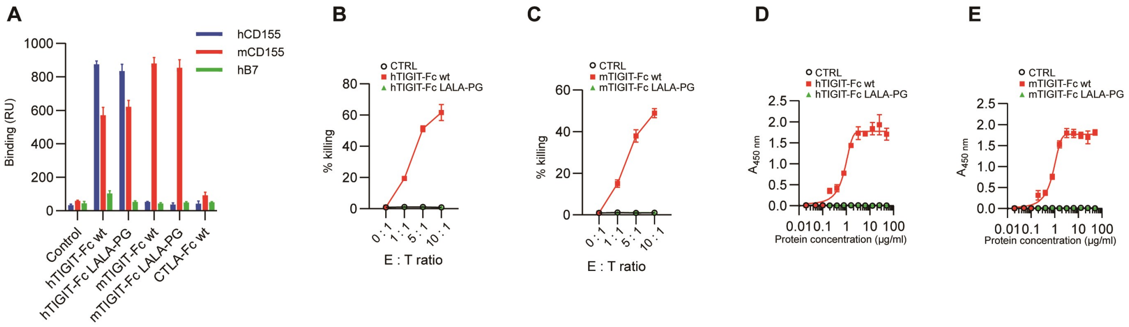

Figure S2. Characterization of TIGIT fusion proteins. (A) Binding of fusion proteins to human antigens. Fusion proteins was tested for binding to immobilized hCD155, mCD155 or B7 protein using surface plasmon resonance on a BIAcore 2000 instrument. Binding was quantified as an increase in RU at 60 s after the end of injection compared with a baseline established 20 s before injection, *n* = 4. In vitro ADCC assay with monocyte-derived macrophages and PVR^{+} target cells in the presence or absence of human **(B)** or murine **(C)** TIGIT-Fc fusion proteins, *n* = 4. Human **(D)** and murine **(E)** TIGIT-Fc fusion proteins were coated on an ELISA plate. The complement component C1q was added to the plate, and its retention was detected via anti-C1q antibodies, *n* = 4.

Figure S3

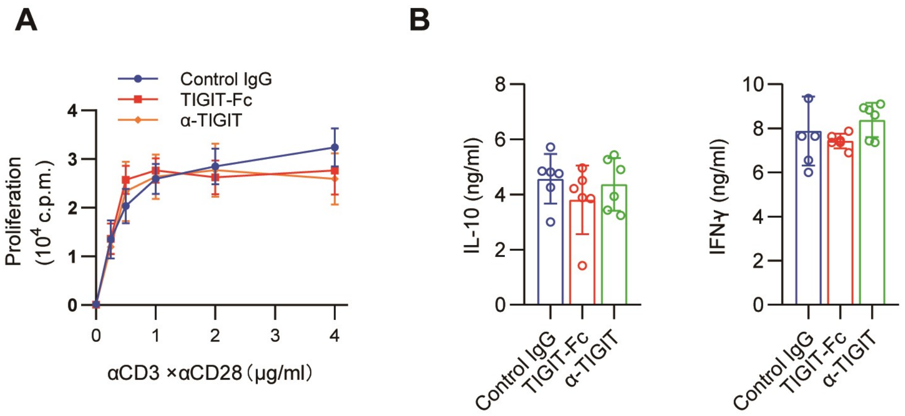

Figure S3. Direct Effect of TIGIT-Fc on human decidua T cells. (A) Proliferation of CD4$^+$CD45RO$^+$ T cells activated with plate-bound anti-CD3 & anti-CD28, together with TIGIT-FC, anti-TIGIT or the control IgG (50 µg/ml), assessed (in triplicate) by [^{3}H]thymidine incorporation on day 4, *n* = 4. **(B)** Luminex assay of cytokines in supernatants of the cultures in A, collected on day 2 with plate-bound anti-CD3 & anti-CD28 (2 µg/ml each).

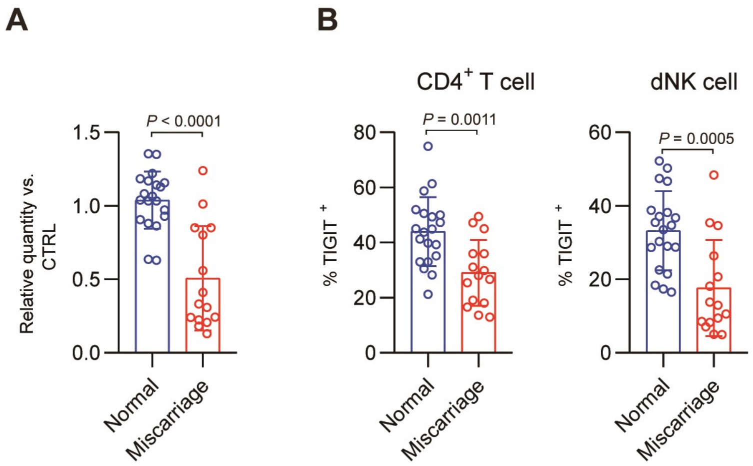

Figure S4. Higher TIGIT expression at maternal-fetal interface in the early pregnancy than miscarriage. (A) Quantitative PCR shows that TIGIT expression in decidua from placenta of normal early pregnancy was significantly higher than that of unexplained miscarriage, *n* = 15-20. **(B)** The proportions of cells with positive TIGIT expression determined by flow cytometry analyses in dCD4^{+} T and dNK cells of normal early pregnancy was significantly higher than that of unexplained miscarriage, *n* = 15-20. Data are the means ± s.d. **(A-B)** and *P* values were from a nonparametric t test **(A-B)**.

Figure S5

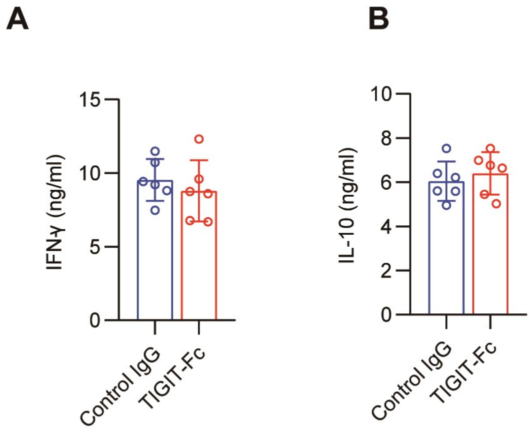

Figure S5. Direct Effect of TIGIT-Fc on mice decidua T cells. Mice decidual $CD4^+$ cells were stimulated with anti-mouse CD3&CD28 together with TIGIT-Fc or the control IgG for 48h and Elisa assay was used to assess INF-γ **(A)** and IL-10 **(B)** level in supernatants of the cultures, $n = 6$.

Supplementary Tables

Table S1. Selected analytical data and pharmacokinetic parameters of recombinant fusion proteins in mice.

Parameter[a]	CTLA4-Fc wt	hTIGHT-Fc wt	hTIGIT-Fc LALA-PG	mTIGIT-Fc wt	mTIGIT-Fc LALA-PG
HMW formation after storage(% SEC area)[b]	< 0.1	< 0.1	< 0.1	< 0.1	< 0.1
LMW formation after storage(% SEC area)[b]	< 0.1	< 0.1	< 0.1	< 0.1	< 0.1
AUC (day µg ml^{-1})	492.20	524.63	489.03	517.63	495.33
$T_{1/2}$(day)	5.82	6.66	4.90	4.95	5.91
CL (ml day^{-1} kg^{-1})	7.31	6.98	7.97	7.72	7.68
VSS(ml kg^{-1})	81.14	77.43	77.35	70.83	78.61

[a] Pharmacokinetic parameters were calculated using a noncompartmental analysis. AUC, area under the concentration versus time curve; t1/2, half-life; CL, clearance; VSS, steady-state volume of distribution.

[b] Quiescent storage for 3 wk, 40 °C, 1 mg/mL

Table S2. Details of compound library in each plate

Axitinib	Tizanidine HCl	Gefitinib (ZD1839)	Tipifarnib	Sorafenib Tosylate	Daclatasvir (BMS-790052)	Crizotinib (PF-02341066)	Betamethasone	Docetaxel	Natamycin
Vemurafenib (PLX4032, RG7204)	Ketorolac	Benazepril HCl	Enalaprilat Dihydrate	Tegafur (FT-207, NSC 148958)	Aminoglutethimide	Edaravone	Ipratropium Bromide	Fluvoxamine maleate	Hydrocortisone
Roxadustat (FG-4592)	Topiramate	Imatinib Mesylate (STI571)	Atazanavir Sulfate	Sunitinib Malate	Iloperidone	Vismodegib (GDC-0449)	Mycophenolate Mofetil	Paclitaxel	Telaprevir (VX-950)
Acarbose	Adenosine	Budesonide	Dofetilide	Ifosfamide	Aminophylline	Etodolac	Sulfanilamide	Gatifloxacin	Desonide
Nintedanib (BIBF 1120)	Tranilast	Lapatinib (GW-572016) Ditosylate	VX-745	Temsirolimus (CCI-779, NSC 683864)	HMN-214	Belinostat (PXD101)	Dyphylline	Capecitabine	Saxagliptin
Adapalene	Zolmitriptan	Bumetanide	Isradipine	Megestrol Acetate	Lubiprostone	Etomidate	Betamethasone Dipropionate	Genistein	Didanosine
Afatinib (BIBW2992)	Venlafaxine HCl	Lenalidomide (CC-5013)	Moxifloxacin HCl	Vorinostat (SAHA, MK0683)	Naratriptan HCl	Rucaparib (AG-014699,PF-01367338) phosphate	Aztreonam	Lenvatinib (E7080)	Febuxostat
Altretamine	Telbivudine	Carmofur	Estrone	Mercaptopurine (6-MP)	Amorolfine HCl	Felbamate	Meprednisone	Glimepiride	Divalproex Sodium
Anastrozole	Pomalidomide	Cladribine	Olmesartan Medoxomil	Methotrexate	Silodosin	Letrozole	Naproxen Sodium	Entecavir Hydrate	Amphotericin B
Loratadine	Deferasirox	Ruxolitinib (INCB018424)	Azathioprine	Omeprazole	Meloxicam	Tenofovir Disoproxil Fumarate	Nevirapine	Clopidogrel	Pitavastatin Calcium
Aprepitant	Tazarotene	Decitabine	Cefdinir	Bendamustine HCl	Riluzole	Temozolomide	Nitazoxanide	Nepafenac	Ibuprofen
Losartan Potassium (DuP 753)	Piroxicam	Isotretinoin	Indomethacin	Ondansetron HCl	Mesna	Tenofovir	NEXIUM (esomeprazole magnesium)	Ranolazine 2HCl	Rifapentine
Bicalutamide	Fasudil (HA-1077) HCl	Dutasteride	Clotrimazole	Epirubicin HCl	Sulfameter	Tamoxifen	Triamcinolone Acetonide	Rufinamide	Amprenavir
Amonafide	Gemcitabine	Lopinavir	Terbinafine	Oxcarbazepine	Methocarbamol	Tigecycline	Nicotinic Acid	Repaglinide	Suprofen
Fulvestrant	Sulfasalazine	Melatonin	Rizatriptan Benzoate	Etoposide	Prilocaine	Vincristine sulfate	Orlistat	Posaconazole	Albendazole
Acitretin	Glipizide	Meropenem	Levonorgestrel	Pirarubicin	Prednisolone	Trilostane	Nimodipine	Rolipram	Pyrazinamide

Bortezomib (PS-341)	Voriconazole	Panobinostat (LBH589)	Calcitriol	Entinostat (MS-275)	Ponatinib (AP24534)	Cabozantinib (XL184, BMS-907351)	Alprostadil	Valproic acid sodium salt (Sodium valproate)	Dapagliflozin
Amisulpride	Monobenzone	Cetirizine DiHCl	Flucytosine	Streptozotocin (STZ)	Chloramphenicol	Fluconazole	Betamethasone Valerate	Ivermectin	Emtricitabine
Bosutinib (SKI-606)	Zileuton	Nilotinib (AMN-107)	Doxercalciferol	Enzastaurin (LY317615)	Fludarabine	Everolimus (RAD001)	Lactulose	Regorafenib (BAY 73-4506)	Nebivolol HCl
Aniracetam	Tretinoin	Cilnidipine	Trichlormethiazide	Costunolide	Flurbiprofen	Flumazenil	Praziquantel	Lansoprazole	Progesterone
Dasatinib	Ziprasidone HCl	Pazopanib HCl (GW786034 HCl)	Alfacalcidol	Olaparib (AZD2281, Ku-0059436)	Pralatrexate	Malotilate	Tadalafil	Danoprevir (ITMN-191)	Pimobendan
Artemisinin	Phenylbutazone	Cilostazol	Loteprednol etabonate	Dexamethasone (DHAP)	Disulfiram	Fluoxetine HCl	Busulfan	Levetiracetam	Lamivudine
Ridaforolimus (Deforolimus, MK-8669)	Zonisamide	Rapamycin (Sirolimus)	Safinamide Mesylate	Masitinib (AB1010)	Cefaclor	Ivacaftor (VX-770)	Cyclosporine	Ritonavir	VX-809 (Lumacaftor)
Asenapine maleate	Ezetimibe	Floxuridine	Nirogacestat (PF-03084014)	Doxazosin Mesylate	Mesalamine	Flupirtine maleate	Carbamazepine	Lidocaine	Estradiol
Raltitrexed	Candesartan	Bisoprolol fumarate	Pyridostigmine Bromide	Raloxifene HCl	Darunavir Ethanolate	Agomelatine	Allopurinol	Prasugrel	Chlorothiazide
Daptomycin	Glyburide (Glibenclamide)	Mianserin HCl	Gemfibrozil	Pizotifen Malate	Telmisartan	Vecuronium Bromide	Nisoldipine	Sildenafil Citrate	Quetiapine Fumarate
Thalidomide	Apixaban	Tivozanib (AV-951)	Methimazole	Fludarabine Phosphate	Prednisone	Leflunomide	Allopurinol Sodium	Ramelteon	Ursodiol
Doripenem Hydrate	Fomepizole	Mosapride Citrate	Indapamide	Resveratrol	Thiabendazole	Bimatoprost	Octocrylene	Sumatriptan Succinate	Rifampin
Exemestane	Reserpine	Doxorubicin (Adriamycin) HCl	Metolazone	Topotecan HCl	Acetylcysteine	Enzalutamide (MDV3100)	Zafirlukast	Cinacalcet HCl	Nitrofural
Gestodene	Adefovir Dipivoxil	Nafamostat Mesylate	Mitotane	Rocuronium Bromide	Guaifenesin	Linezolid	Oxybutynin	Tamsulosin	Cefditoren Pivoxil
Finasteride	Furosemide	Fluorouracil (5-Fluoracil, 5-FU)	Cefoperazone	2-Methoxyestradiol (2-MeOE2)	Ethinyl Estradiol	Dienogest	Erythromycin	Celecoxib	Ketoprofen
Drospirenone	Zalcitabine	Naftopidil DiHCl	Methylprednisolone	Stavudine (d4T)	Rifabutin	Alfuzosin HCl	Enoxacin	Tianeptine sodium	Sulfadiazine
